# Regulation of diverse nuclear shapes: pathways working independently, together

**DOI:** 10.1080/19420889.2021.1939942

**Published:** 2021-07-05

**Authors:** Pallavi Deolal, Krishnaveni Mishra

**Affiliations:** Department of Biochemistry, School of Life Sciences, University of Hyderabad, Hyderabad, India

**Keywords:** Nucleus, nuclear shape, nuclear envelope, morphology, nuclear pore complex, lamins, nuclear organization

## Abstract

Membrane-bound organelles provide physical and functional compartmentalization of biological processes in eukaryotic cells. The characteristic shape and internal organization of these organelles is determined by a combination of multiple internal and external factors. The maintenance of the shape of nucleus, which houses the genetic material within a double membrane bilayer, is crucial for a seamless spatio-temporal control over nuclear and cellular functions. Dynamic morphological changes in the shape of nucleus facilitate various biological processes. Chromatin packaging, nuclear and cytosolic protein organization, and nuclear membrane lipid homeostasis are critical determinants of overall nuclear morphology. As such, a multitude of molecular players and pathways act together to regulate the nuclear shape. Here, we review the known mechanisms governing nuclear shape in various unicellular and multicellular organisms, including the non-spherical nuclei and non-lamin-related structural determinants. The review also touches upon cellular consequences of aberrant nuclear morphologies.

## 1. Introduction

The nucleus is one of the key organelles that distinguishes eukaryotes from prokaryotes. The nucleus contains the genetic material and is surrounded by the double-membraned nuclear envelope (NE). The NE is perforated by multi-protein assemblies referred to as nuclear pore complexes (NPCs) at sites where the inner and outer nuclear membranes fuse. They serve as the sites of exchange of material between the cytoplasm and nucleus. The NE is more than an inert physical barrier; it coordinates functions like DNA replication, transcription, nucleo-cytoplasmic transport, nuclear division, and protein quality control. Specialized proteins in the inner nuclear membrane (INM) associate with chromatin to regulate nuclear structure and function. In many animals, a mesh-like layer of the nuclear lamina, comprising members of the intermediate filament family, the lamins, underlies the INM. A unique protein composition differentiates the outer nuclear membrane (ONM) from the otherwise contiguous endoplasmic reticulum (ER). In some species where the nucleus undergoes closed mitosis, the NE also houses the spindle pole body (SPB), which forms attachments to the kinetochore-centrosome complexes of the chromosomes.

The interior of the nucleus is structurally and functionally compartmentalized, but these compartments are not membrane-bound. The most prominent visible feature of a nucleus is the nucleolus; some cells have more than one nucleoli. Each nucleolus is a functional compartment, which is the site for transcription and processing of ribosomal RNA (rRNA) and assembly of ribosomes. Genes encoding rRNA are organized in tandem repeats; these rDNA loci may lie on multiple locations on the genome. All rDNA loci converge in the nucleolus. In budding yeast, the nucleolus appears as a peripheral crescent-shaped structure occupying almost one-third of the nucleus. In mammals, nucleoli lie close to the center of the nucleus [1]. Other distinct compartments include the transcription and pre-mRNA splicing sites which localize in the form of numerous discrete dots distributed throughout the nucleus. The chromosomes are also organized nonrandomly and in most cell types, the heterochromatin is localized toward the nuclear periphery and the euchromatin is more interior from the nuclear periphery [2].

The function of an organelle in a cell is linked to its structure and overall organization of its sub-compartments. Improper spatial placement and altered levels of essential skeletal proteins affect not only the normal geometry, but often even the function, of an organelle. Likewise, nuclear function is also integrated with changes in its morphology, which is in turn responsive to cellular processes such as growth, development, aging, and movement. Conversely, functional defects in the proteins of the nuclear matrix and INM are associated with aberrations in nuclear organization [3,4], and lamins [5]. Atypical nuclear shape is observed in many pathological conditions, such as cancer, several striated muscle laminopathies, Hutchinson-Gilford Progeria Syndrome, and mandibuloacral dysplasia [6–9]. Thus, there appears to be a correlation between morphological changes in the nucleus and the function of the nucleus.

While it is clear that abnormalities in nuclear shape are associated with pathological states, our understanding of mechanistic basis of nuclear organization and maintenance of nuclear shape is weak. In this review, we discuss the diversity in nuclear shapes across organisms and the factors involved in maintenance of morphology. Following this, we give an account of known physiological and pathological states associated with altered nuclear shape to call attention to nuclear shape regulation. Some of the outstanding questions in the field of shape regulation and function are also surmised.

## 2. Diversity in nuclear shape

Even though nuclei are often represented as spherical/round organelles, deviations from this morphology are seen across the eukaryotic supergroups. Shapes as diverse as ovoid, lobed or condensed nuclei are seen in mammalian cells [reviewed by 10]. For instance, the nuclei of granulocytes and monocyte lineage of the mammalian immune system are multi-lobed or kidney-bean shaped while lymphocytes have a spherical, round nuclei [11,12]. Heterophils, the equivalent of neutrophils in birds, show species-specific differences in the presence or absence of nuclear lobes [13]. Lobed nuclei have their genetic material packaged in more than one sphere-like organization giving the NE a lobulated appearance [14]. Multiple lobes are often connected with a thin chromatin-containing filamentous structure. Spermatozoa also exhibit wide variations in nuclear shapes in animals [15]. The highly condensed sperm nucleus has asymmetric shapes depending on the shape of the sperm head since the nucleus occupies most of the sperm head. Most mammalian sperms including those of whales and dolphins have oval or paddle-shaped nuclei whereas birds display either tubular or worm-like nuclei [15–17].

Plants too exhibit a tissue-specific diversity of nuclear shapes [reviewed by 18]. Spindle-shaped nuclei are seen in differentiated root epidermal and cortical cells [19]. Meristematic and vascular tissues have spherical and rod like nuclear shapes, respectively [20]. Nuclei may have grooved surfaces, as in epidermal cells in onion and invaginations of NE produce lobed nuclei [21].

**Figure 1.**
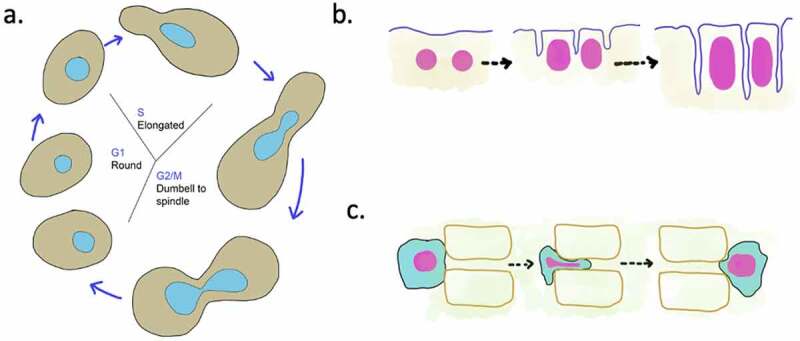
Nuclear shape changes during cellular processes

Nuclear morphology may change during physiological states such as nuclear division, nuclear fusion during mating, development, differentiation, and cell migration (Figure 1). For instance, the round nuclei of budding yeast become dumbbell or spindle-shaped as they transition from S phase to G2, M [22] (Figure 1(a)). During the plant root development, cellular elongation is accompanied by elongation of nuclei [23]. In *Drosophila*, the change of spherical to elliptical nuclear shape is critical during the cellularization stage, wherein asynchronous cytokinesis in the syncytial blastoderm leads to formation of uninucleate cells [24] (Figure 1(b)). This defined increase in nuclear size and change in shape is crucial for the process of development [24]. Thus, it appears that maintenance of cell-type-specific nuclear morphology is highly regulated. In the following section, we discuss some of the components known to regulate nuclear shape.

a. Changes in the nuclear morphology is coordinated with the yeast cell cycle. Yeasts (brown) that undergo closed mitosis display a round to oval to spindle shaped nuclear shape (cyan) changes before dividing into two round nuclei again.

b. During larval development in *Drosophila*, the nuclei (magenta) grow in size and change from spherical to elliptical shape. This nuclear shape change is an important part of cellularization.

c. Cells (cyan) that migrate undergo nuclear shape (magenta) deformation while passing through constricted tissue spaces. Failure to modulate the nuclear shape can slow migration or lead to loss of nuclear integrity and cellular damage. Few examples of nuclear shape changes associated with migration are during plant root hair development, immune response in humans and tumor metastasis.

## 3. Regulators of nuclear shape

Of the various internal and external factors that determine nuclear shape, studies have reported the roles of genome size and ploidy, functional requirements of the cell, lifespan, chemical environment, and signaling. We discuss below some of these factors. While the main focus will be on nuclear shape, known connections between the size and shape of the nucleus are also discussed.

### a. Chromatin

The amount of chromatin can be expected to be a key determinant of nuclear shape and size across organisms. Although the nuclear size is not strictly dependent on the DNA content *per se*, ploidy differences do induce nuclear shape changes during development [18,24,25]. The architecture and packaging of chromatin contributes significantly to nuclear morphology and firmness, as evidenced by studies in budding yeast (*Saccharomyces cerevisiae*), fission yeast (*Schizosaccharomyces pombe*), and human cells [26–28]. Nuclei of *S. cerevisiae* have a Rabl-like arrangement of chromosomes, with the centromeres clustering to one end of the nucleus and the telomeres tethered to the NE at distances dictated by the chromosome arm lengths [29,30]. The chromosomes organize along the central nuclear axis from the spindle pole body (SPB) to the diametrically opposite nucleolar center [31]. In the single nucleolus, the rDNA is tethered to the INM differently than bulk DNA via association with specific proteins of the INM [32].

Among plants, organisms like *Arabidopsis thaliana* and *Zea mays* display a non-Rabl like organization, while species like *Triticum aestivum* and *Avena sativa* have Rabl-like chromosome configuration [33]. Interestingly, in both the classes, INM proteins are involved in anchoring telomeres to the periphery. Attachment of chromatin to the NE provides a mechanical link between the components of the nucleus and provides rigidity and mechanical strength to the nucleus [27,34].

**Figure 2.**
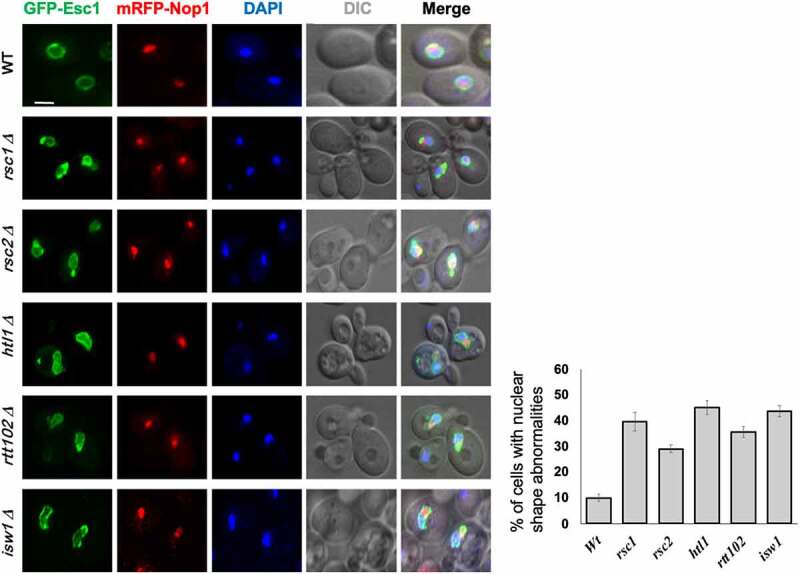
Loss of non-essential members of RSC chromatin-remodeling complex affects nuclear shape in *S. cerevisiae.*

The role of ATP-dependent chromatin remodelers in restructuring nucleosomes is well known [35]. As chromatin is tethered to the NE via INM proteins, loss of components of chromatin remodeling complexes (SWI/SNF1 in humans and RSC in *S. cerevisiae*) perturbs nuclear morphology [28,36]. Absence of Brg1 in the SWI/SNF complex leads to invaginations of NE in breast cancer epithelial cells in [36]. It is proposed that the Brg1 protein (and other chromatin remodelers) affects nuclear shape by altering chromatin structure: a reduction or loss of activity of remodelers causes alterations in nucleosomal organization wherein increased condensation of chromatin “pulls” the NE inward due to the tethering of chromatin to NE [36]. A study which examined the organization of NPCs in some of the chromatin remodeling mutants also reported changes in the shape of budding yeast nucleus [28]. The mutants had abnormal NE morphology as well as mislocalization of NPCs. Interestingly, the addition of benzyl alcohol, an agent that increases membrane fluidity, restores the nuclear shape in these mutants, indicating mechanical reasons behind the shape changes [28]. While this study examined most of the essential genes of the RSC complex, we tested the components of the RSC and ISW (imitation switch) class of chromatin remodelers encoded by the non-essential genes. We found that these mutants also have abnormal nuclear morphology in *S. cerevisiae* (unpublished data; Figure 2).

Live cell imaging from an unsynchronized culture of the indicated strains transformed with plasmids expressing GFP-Esc1 (nuclear membrane marker) and mRFP-Nop1 [nucleolar marker) was done to examine the nuclear and nucleolar morphology asdescribed in 41. DAPI was used to stain the nuclear DNA. The fraction of cells showing abnormal nuclear shape in each of the mutants was quantified from ~200 cells from 2 independent experiments and is shown in the bar graph. Scale-2 µm

Defective chromatin condensation and altered distribution of heterochromatin also affect nuclear shape in mammals and plants. Failure to condense chromatin in mice is associated with misshaped sperm heads that lack the typical hook-like head [37]. Double mutants of mammalian H3K9me1 methyltransferases Prdm3 and Prdm16 result not only in the loss of Lamin A/C at the NE but also in invaginations of NE [38]. Mutants of nuclear envelope associated proteins (*neap1 neap3)* display nuclear shape change concomitant with alterations in nuclear organization and heterochromatin condensation in guard cells of *Arabidopsis thaliana* [39]. While in these cases it appears that chromatin organization *per se* in the nucleus contributes to normal shape of the nucleus, the possibility that these architectural defects are due to change in expression of structural genes cannot be ruled out.

### b. Lamins and similar INM proteins

#### Lamins

Attachment of chromatin to lamins and INM proteins provides another layer of regulation for genome organization and the basis for mechanical changes in the NE. In budding yeast, the association of chromatin with NE proteins like Esc1, Mps3, Heh1 and Heh2, means that a change in chromosome structure could potentially affect the NE by altering the tethering [22,40–42]. The role of lamins and other NE proteins in nuclear shape regulation will be discussed in the following sections.

Lamins are intermediate filament class of cytoskeletal proteins known to be crucial for maintaining nuclear shape. Structural homologues of lamins are found in metazoans, while functional homologues are proposed to exist in other supergroups like plants and ameobae. Lamins are involved in a multitude of nuclear functions such as supporting the nuclear structure, regulating genome organization, DNA repair and transcription regulation, and nuclear mechanics [43]. Humans have two types of lamins- Lamin A and Lamin B. Lamin A and lamin C are alternately spliced *LMNA* gene products, whereas Lamin B1 and B2, are expressed from the gene *LMNB1* and *LMNB2* respectively [43].

The expression of various classes and isoforms of lamins is highly specific to a cell and organism and has a bearing on the nuclear organization [44] . Low lamin A/C is responsible for lobulation of neutrophil nuclei [11]. Loss of function mutation in lamin A leads to premature aging disease, Hutchinson’s progeria [45]. A striking feature of this condition is the deformed nucleus with lobulated NE. Interestingly, similar nuclear shape alterations are seen in physiologically aged nucleus suggesting a correlation between nuclear morphology and aging [46]. While it is not clear if nuclear shape alteration is the cause or consequence of the disease, expression of defective laminA causes nuclear deformities [recently reviewed in 47]. Dysfunction of *Drosophila* lamin Dm_0_ expressed early in development causes multiple organizational defects of the nucleus [48]. In *C. elegans*, reduced expression of lamin gene causes abnormalities such as asymmetric nuclear division and rapid changes in nuclear shape in early embryogenesis [49].

#### Lamin-like proteins

While fungi and plants lack proteins with extensive homology to lamins, they do have several INM proteins that resemble lamins either structurally or functionally. Lamin-like nuclear coiled-coil proteins in plants were first identified in carrot, *Daucus carota* [50,51] . This class of proteins encoded by CRWN (crowded nucleus) genes is functionally analogous to lamins [52] and mediates chromatin tethering to the nuclear periphery [53]. *crwn* mutant nuclei are smooth with no invaginations but are more deformable. This is perhaps because the tethering of centromere and other heterochromatic regions to the nuclear periphery through CRWNs is disrupted and makes the nuclei more malleable [34,53].

Sequence-based prediction studies have also identified the presence of lamin analogues NMCP1 and NMCP2 (Nuclear Matrix Constituent Proteins, encoded by CRWN) in nuclei of monocot *Allium cepa*, onion [54,55]. AcNMCP1 and AcNMCP2 show an overlapping distribution along the nuclear periphery, encircling the chromatin, in a manner similar to mammalian lamins. This distribution changes slightly, becoming discontinuous, during root differentiation [54]. NMCP1 and NMCP2 are currently the most-likely candidates for lamin homologues in plants [56]. In *Arabidopsis* too, the CRWN1-4 appear important for nuclear shape and size regulation [53,56]. *crwn* mutants have smaller nuclear volume (hence also referred as *linc-* little nuclei mutants) with increased sphericity compared to the wild type cells though there is no effect on the gene regulatory mechanisms [20].

Other groups of lamin-like NE proteins in lower eukaryotes are also distinguished by the presence of coiled-coil motifs at their C terminal domain. One such protein is the *Kugelkern* (*Kuk*) protein of *Drosophila*; increased abundance of *Kuk* is associated with nuclear elongation and formation of lobes and wrinkles [24]. In the tunicate *Ciona*, two lamin variants L1 and L2, lack a 90-amino acid domain at their globular ‘tail’ region, but bear homology at the alpha-helical domain [57,58]. Another coiled-coil domain containing protein is the INM protein Esc1 of *S. cerevisiae*, which is involved in anchoring of telomeres [59]; over-expression of Esc1 produces abnormally long extensions of the NE [41,60]. While Esc1 is restricted to Saccharomycetes, the *S. cerevisiae* proteins Ebp2 and Rrs1 falling in this group are universally conserved and have key roles in anchoring and clustering of telomeric heterochromatin, and in ribosome biogenesis [61,62]. Loss-of-function mutations of these proteins that do not affect ribosome biogenesis, disrupt telomeric gene silencing and clustering without affecting anchoring to NE, indicating links between NE proteins, nuclear shape and chromatin function.

Lamin-like proteins are also seen in Dinoflagellates and *Trypanosoma* [63–65]. A lamin-like protein, NE81 has been studied in *Dictyostelium* [66]. Null mutants of NE81 display misshaped nuclei and abnormal chromosome distribution. Wild type and overexpressed NE81 localizes to the NE like mammalian lamins and upon ectopic expression in mammalian cells, NE81 colocalized with lamin B1 [66]. The NUP-1 protein of *Trypanosoma brucei* locates to the NE and regulates gene expression, heterochromatin establishment and nuclear shape [63]. As in lamin mutants, knockdown of NUP-1 results in the loss of NE integrity, disturbed NPC distribution and chromosomal organization. The fresh water filasterean *Capsaspora owczarzaki* has a protein similar to human Lamin B, although its effect on nuclear shape and organization is not known [67,68] The contribution of these lamin-like proteins to organization, mechanical stability and shape of nucleus merit deeper examination to uncover the evolution of this class of proteins and their role in nuclear organization.

#### Lamin-interacting proteins

Many INM proteins interact with lamins via a helix-extension-helix motif containing LEM domain (LAP2-Emerin-MAN1); these proteins function in chromatin attachment and impart structural stability to the nucleus [69–72]. Homologs of LEM domain containing proteins have been identified in organisms across all super groups [61,73]. In *S. cerevisiae*, the LEM domain proteins, Heh1 (also known as Src1) and Heh2 regulate the NPC quality [74,75]. Heh1 contributes to NPC stability by interacting with membrane nucleoporins (nups) such as Pom152 at the NE lumen [76]. Loss of *HEH1* does not cause any NE deformations and nuclei remain spherical, although are larger in size [41,77]. In *S. pombe*, LEM domain proteins Lem2, Man1 and Ima1 are involved in heterochromatin silencing at rDNA, centromeric and telomeric loci [69]. These functions of Man1 and Lem2 are considered similar to that of lamins in metazoans. Absence of *MAN1* and *LEM2* causes blebbing of NE and discontinuous membrane distribution [78]. Turning off the expression of Lem2 in the absence of its interactor Bqt4, an abundant INM protein that associates with telomeres in *S. pombe*, leads to loss of NE integrity and aberrant nuclear shape [79,80]. Deformations of the NE have also been observed in the absence of *IMA1*. Nuclei of *S. pombe ima1∆* cells are ovoid rather than spherical and display ‘ruffled’ NE [77]. The *C. elegans* genome encodes four distinct LEM proteins: EMR-1, LEM-2, LEM-2, and LEM-4. Owing to their overlapping functions, *lem-2*, but not *emr-1* mutants show nuclei with reduced circularity and invaginated NE [81].

#### Nucleo-cytoskeletal complex

A wide spectrum of protein-protein interactions at the nuclear periphery contribute to the stability of nucleus and shape maintenance. The association of nuclear and cytosolic elements is mediated by the linker of nucleoskeleton and cytoskeleton (LINC) complex. SUN (Sad1-UNC-1)- and KASH (Klarsicht-Anc-1-Syne-1)- domain containing proteins mediate the crosstalk from the nuclear and cytoplasmic face respectively. The members of LINC complex are widely conserved from yeast to mammals [61,82]. The role of LINC components in providing shape and stability to the nucleus is well established and extensively reviewed [34,83,84]. Disrupting LINC components leads to distorted nuclear shape and nuclear organization, a property attributed to the deranged cytoskeletal forces acting on the nucleus [85,86].

*D. discoideum* Sun1-Interaptin form the SUN-KASH pair that regulates nuclear positioning and nuclear shape [87]. Sun-1 depletion or expression of a truncated version results in nuclear expansion and appearance of blebs. Deformation of nuclear shape and extensions of NE are reported upon overexpression of mutant *MPS3*, the SUN-domain protein of *S. cerevisiae* [88]. This abnormality is a result of modulation of NE lipid composition. Cells expressing mutant mammalian KASH protein *nesprin-2* display NE abnormalities and blebbing similar to lamin mutants [89]. In *A. thaliana*, loss of SUN1 and/or SUN2 results in circular nuclei of root hair cell [90]. The interaction between AtSUN1/2 at the INM and WPP domain-interacting protein (WIP) at the cytosol forms the LINC complex [91]. AtWIPs also function in anchoring the Ran-GTPase activating protein 1 (RanGAP1) the NE. Together this SUN1/2-WIP1 and WIP1-RanGAP1 interaction is important for maintaining the elongated nuclear shape in leaf epidermal and root hair cells of *A. thaliana* [91]. AtSINE1-4 are other putative KASH domain proteins found at the NE that show tissue-specific expression and differ widely in their conservation status in land plants [92]. AtTIK (*A. thaliana* Toll-Interleukin-Resistance -KASH) is another root cells-specific NE protein that contains a putative KASH domain and controls nuclear morphology [93]. These observations indicate that distinct proteins interact with plant SUN domain family of proteins and together regulate nuclear morphology.

### c. Protein complexes at the NE

The NE acts not only as an enclosure for the nuclear content but also provides a scaffold for protein assemblies that contribute to the overall nuclear architecture. Some of these complexes participate in the formation of membrane curvature at the NE and introduce heterogeneity by creating diffusion barriers to lipid movement [94]. These protein complexes also help in regulating NE integrity in unicellular organisms [95].

One of the largest and dynamic protein macro assembly that spans the NE is the SPB [96]. In fungi that undergo closed mitosis, the nucleus displays a series of morphological alterations coordinated with cell cycle stages. In such cells, the SPB is located inside the nucleus and undergoes lateral expansion [97]. During mitosis, the SPB divides and the two spindles move to opposite poles within the NE, pulling the attached chromosomes with them. Normally, the round nucleus elongates to a spindle shape and appears like a dumb-bell right before splitting into two round daughter nuclei. Similar to cytoskeletal and nucleoskeletal components, microtubules radiating from the SPB exert a force on the NE. Thus, the SPB dynamics affects nuclear shape and size [98]. When spindles fail to separate normally, the NE deforms [99]. Excessive elongation of spindle due to defective microtubule-SPB attachment results in the formation of panhandle-shaped protrusions of the NE. In *S. pombe*, for example, the absence of the Dis1p protein, which is required for kinetochore/microtubule attachment results in abnormally deformed nuclei [99]. The *pim1-d1* mutant has defective Ran-GTPase associated with the SPB; this interferes with symmetric nuclear division and results in oblong-shaped nuclei. Mutants of kinetochore proteins Ndc80, Spc24, and Mis17 also show nuclear shape defects associated with nuclear division in *S. pombe* [100].

NPCs are other large multi-protein assemblies inserted into the NE. NPCs comprise multiple copies of around 30 different nucleoporins (nups). The *de novo* assembly of an NPC requires INM and ONM fusion and as such the process is inextricably linked to the NE architecture [101–103]. Nups with membrane binding regions play crucial roles in insertion and stabilization of NPC at the site of pore formation. ScNup1 and ScNup60 support the INM via their amphipathic domains; overexpression of these nups induces membrane curvature and distortions in the NE [104]. The membrane binding regions of nucleoporins (Nup133, Nup53, Pom121, gp210) contribute significantly to the NE stability [105]. Upon silencing of a transmembrane protein gp210 in HeLa cells, along with clustered NPCs, aberrant nuclear membrane structures are observed [106]. Another vertebrate transmembrane nup, Pom121, also contributes to nuclear membrane stabilization and assembly of functional NPCs by regulating the spacing between INM and ONM during fusion to nucleate NPC assembly [107,108].

The distortion of nuclear shape observed in overexpression, complete loss or downregulation of nups further underscores the importance of NPCs in nuclear shape maintenance. For instance, depletion of ELYS/Mel28, a nucleoporin essential for post-mitotic NPC assembly, results in aberrant distribution of lamin A/C and other INM proteins in HeLa cells [109]. Similarly, reduced expression of Nup153 in HeLa cells results in nuclear membrane invaginations and lobulation, and punctate lamin A/C distribution [110]. Depletion of Nup53 or Nup93 in HeLa cells also results in abnormally shaped nuclei albeit without affecting lamin distribution along the NE [111]. Budding yeast cells lacking nups or with other defects in NPC assembly show herniations of the NE [28,74,112]. The overexpression of Nup53 in *S. cerevisiae* leads to the formation of intranuclear double-membrane lamella, which is lined beneath the INM [113]. In *Arabidopsis*, cells lacking the nucleoporin Nup136 show spherical instead of normal ellipsoid nuclei [114]; these mutants have a shorter major axis length compared to the elongated nuclei of wild type. Plant cells that overexpress Nup136 display extremely elongated nuclear structures instead [115]. This shows that the levels of Nup136 are critical for the regulation of nuclear morphology in *Arabidopsis*.

Many nuclear and ER proteins that are not bonafide nucleoporins also influence NPC organization and function, and are characterized by abnormalities in nuclear shape. For example, INM component of the LINC complex, Sun1 is closely associated with NPCs and HeLa cells depleted of Sun1 display NPC clustering with abnormally shaped nuclei [85]. As discussed in the earlier section, loss of chromatin remodelers in *S. cerevisiae* also results in clustering of NPCs along the NE, with accompanying nuclear shape abnormalities [28]. Further, perturbations of NE morphology and NPC clustering can be seen in budding yeast cells lacking reticulons- Rtn1 and Yop1, ER proteins that regulate membrane curvature [116]. Levels of reticulons are crucial for regulated NE expansion and NPC insertion during NE reformation in post-mitotic cells as well [117].

Dysfunctional torsin, a NE associated non-canonical AAA-ATPase, results in abnormal NPC distribution and blebbing of NE in neuronal tissues where it is highly expressed [118]. Nuclear blebbing also occurs in HeLa cell lines lacking torsin and its cofactors [119]. *C. elegans* and mammalian cells expressing mutant torsin also show nuclear blebbing [120,121]. No sequence-homologues of torsin have been reported in lower eukaryotes like yeast; other proteins with similar function may exist in these organisms.

Defective surveillance of NPC assembly pathways can also results in distorted nuclei. Reduced abundance of Vps4, a AAA-ATPase component of endosomal sorting complexes required for transport-III (ESCRT-III), during replicative aging in budding yeast correlates with declined quality control of NPC assembly and nuclear herniations [4]. In *S. pombe* also, nuclei of *vps4∆* cells have severe morphological defects and are leaky due to membrane fenestrations [122]. This is suggestive of association between NPC assembly and nuclear morphology. Under certain circumstances such as those resulting in formation of leaky pores, NE remodeling and shape changes help in mitigating the consequences by sealing the pores [4,75,123].

In sum, large protein complexes such as SPB and NPCs stabilize the overall nuclear structure and membrane curvature by interacting with various proteins including, but not limited to, lamina and members of LINC complex [78,85,99,124]. While it remains to be resolved if the distortions are a primary outcome of subunit depletion, it is also possible that the nuclear shape changes are a secondary consequence of the effect of altered complex stoichiometry on nuclear size and the interaction of members with other structural determinants.

### d. Lipid homeostasis and properties of the NE

Lipids are critical in determining the morphology of a membrane bound organelle. The NE is a highly dynamic, fluidic, and metabolically active enclave [125,126]. The NE expands throughout interphase in proliferating cells and in nuclei undergoing closed mitosis, the NE also expands significantly during anaphase as the spindle elongates [127]. The expansion of NE is dependent on phospholipid biosynthesis; defects in these pathways leads to misshaped, less round nuclei in fission yeast [128]. Phosphatidic acid is the precursor for synthesis of both membrane and storage lipids at the NE/ER and nutrient availability is a key regulator for channeling phosphatidic acid into membrane synthesis or storage in the lipid droplets [128].

Although most of the lipid synthesis is known to take place at the ER surface, recent studies in budding yeast have shown that INM can also act as a metabolically active site for new lipid synthesis [129,130]. Lipid droplets are also present at the INM in addition to ER and could be important for NE remodeling [130,131]. The study by Barbosa et al establishes links between modulation of nuclear shape and lipid storage in *S. cerevisiae* [129]. Translocation of the phospholipid diacylglycerol acyltransferase, ScLro1, from ER to INM results in generation of triacylglycerols (TAGs) and lysophospholipids at this location. At the INM, Lro1 is excluded from the nucleolar territory under conditions of nuclear expansion. Thus, a strict compartmentalization of Lro1 and TAG synthesis at the INM is important for modulation of NE morphology in response to signals that affect lipid biosynthesis.

Deletion mutants of genes regulating membrane lipid homeostasis in yeast have abnormal nuclear phenotype [128,132,133] and dysfunction of either one of these multiple proteins renders the membrane insensitive to fluidity changes, resulting in extensive NE proliferation [134]. In *S. cerevisiae*, mutations of Apq12 which functions in sterol homeostasis in concert with Brr6 and Brl1, leads to abnormal inclusions of membrane toward the NE lumen [135]. Dysregulation of Pah1 (phosphatidic acid phosphohydrolase 1) and Dgk1 (Diacylglycerol kinase 1), key enzymes that regulate membrane biogenesis and lipid storage, respectively, is linked to abnormal proliferation of NE [132,136].

Abnormal structures of the NE in association with nucleolus have been seen in fungi [41,129,137,138] as well as plants [21]. However, an association between abnormal nucleolar domain and dysregulated lipid metabolism has been reported only in *S. cerevisiae* but not plants [41]. Interestingly, nuclear flares in fission yeast *S. pombe* and *Schizosaccharomyces japonicus* are not found in association with nucleolus [128]. Collectively, these data suggest that nuclear compartments may have species-specific effects on nuclear shape. Alternately, it could also mean that there are differences in the sites of new membrane synthesis in organisms. Addition of new membrane components at specialized NE domains versus mostly uniform addition across the NE could result in the differences in nuclear morphology.

### e. Nuclear size

It has been speculated that a nucleus changes shape in order to maintain a constant ratio of nuclear to cell volume [139,140], given that nuclear volume is maintained within a specific range (8–10% of cell volume) in all organisms. However, nuclear volume can be induced to increase under conditions where nuclear growth is not inhibited but cellular growth is restricted [22,141], or where nucleoplasmic content is increased by enhanced nuclear import, decreased export, or alterations in certain NE proteins [142]. In higher organisms, increased nuclear content appears to lead to larger nuclear volume due to isometric expansion of the NE. However, in *S. pombe*, alterations in nuclear transport lead to increased nuclear volume as well as shape defects due to membrane over-proliferation [143]. In this context, we have noted that an increase in nucleolar size in *S. cerevisiae* mutants is often linked with abnormal nuclear morphology [41]. Here, in misshaped nuclei with increased NE surface area, the expansion of NE is often, but not always, restricted to the nucleolar region. Interestingly, this shape change could be overcome by reducing either the nucleolar size (and, by extension, nuclear volume) or the tethering of rDNA to the NE. Loss of tethering did increase nuclear volume, but did not alter nuclear shape. This indicates two points: one, nuclear shape may change in response to expansion of NE in order to accommodate the expanding nuclear volume, and two, if the NE expansion occurs evenly across the NE, nuclear volume may increase without any distortion of shape [139]. These observations suggest that the volume of a nucleus can affect nuclear shape and that the tethering of chromatin to the NE via NE proteins is an important regulator of NE shape, at least in lower eukaryotes.

The various factors discussed in these sections function in coordination to regulate the overall nuclear shape and architecture (Summarized in Table 1, Figure 4). In most cases, shape changes in the nucleus are accompanied by changes in chromatin organization, distribution of NPCs, and gene expression. A better understanding of protein targeting to the INM and protein-protein interaction networks and their interactions with the lipids in the membrane will bridge the knowledge gap between the assembly of protein complexes and regulation of nuclear shape and integrity.

**Table 1.** Summary of components regulating nuclear shape

Organism	Protein	Function		Localization	Associated defect	Reference
*S. cerevisiae*	Esc1	**E**stablishes **S**ilent **C**hromatin, involved in telomere clustering and Sir4-mediated silencing		INM (absent beneath the nucleolus)	Overexpression results in nuclear membrane ‘escapades’ formation associated with nucleolus	[60]
*S. cerevisiae*	Mps3	SPB insertion and duplication		INM, SPB	Overexpression results in membrane proliferation, and a few extensions and protrusions	[88]
*S. cerevisiae*	Acc1	**A**cetyl **C**oA **c**arboxylase, catalyses the first rate-limiting step of *de novo* fatty acid synthesis		Cytoplasm	INM protrudes into the intermembrane space resulting in ‘islands’ between the INM and ONM in the mutants	[168]
*S. cerevisiae*	Spo7	Regulatory subunit of Nem1-Spo7 phosphatase involved in phospholipid biosynthesis, dephosphorylates Pah1			Misshaped nuclei with a single ‘flare’ like protrusion that colocalizes with the nucleolus	[137]
*S. cerevisiae*	Hmg1	Enzyme 3-**h**ydroxy-3-**m**ethyl**g**lutaryl coenzyme A (HMG-CoA) reductase catalyzing a rate-limiting step of sterol biosynthesis		Cytoplasm, Nuclear associated membranes	Proliferation of closely apposed pair of membrane called ‘karmellae’ with some discontinuities	[169]
*S. cerevisiae*	Dgk1	CTP-dependent **D**iacyl **g**lycerol **k**inase involved in phosphatidic acid biosynthesis		NE, ER	Overexpression results in membrane expansion around nucleus making it irregularly shaped and enlarged	[136]
*S. cerevisiae*	Pah1 (also referred as Smp2)	Phosphotidate phosphatase that dephosphorylates PA to yield diacylglycerol		Nucleus, NE/ER	Deletion results in enlarged, irregularly shaped nuclei with interconnected lobes	[132]
*S. cerevisiae*	Nup1, Nup53, Nup60, Nup116 and other Nups	Nucleoporins		NE, NPC	Deletion and/or overexpression is associated with irregular nuclear shape and membrane extensions	[74,104,112,113]
*S. cerevisiae*	Apq12	Functions in lipid homeostasis along with Brr6, Brl1		NE, ER	Membranous divisions within the NE, invaginations and extensions of NE resulting in abnormal shape	[135]
*A. thaliana*	CRWN1/4	**Cr**o**w**ded **n**ucleus proteins, provide lamin-like stability to plant nucleus		INM	Mutants have nuclei more round and soft than Wild type with irregular nuclear margins	[52,170]
*A. thaliana*	CRWN2/3	Provide lamin-like stability to plant nucleus		Nucleus, INM	Result in abnormal phenotype only when combined with CRWN1/4	[56,170]
*A. thaliana*	KAKU4	Required for maintaining spindle shaped nuclei, interacts with CRWN1/4 (kaku- Japanese word for nucleus)		INM	Nuclear membrane invagination and stack formation in smaller and less elongated nuclei	[171]
*A. thaliana*	SUN1/2	SUN-domain containing components of plant LINC complex required for formation and maintenance of polarized nuclear shape in root hairs		INM	Mutants have nearly round nuclei unlike the highly elongated wild type nuclei in mature root hair	[90]
*A. thaliana*	WIP12/3	WIPs **(W**PP domain–**i**nteracting **p**roteins) are plant-specific KASH proteins required for maintaining elongated plant nuclei in epidermal cells		INM	Less elongated nuclei	[91]
*A. thaliana*	SINE1-5, TIK1	Tissue-specific plant KASH domain proteins		INM, NE	Abnormal nuclear morphology and positioning	[92,93]
*A. thaliana*	Nup136	Nucleoporin		NE, NPC	Mutants display abnormally circular nuclei	[114,115]
Mammals	Lamin A/C	Regulate nuclear shape and rigidity, chromatin attachment and interaction with LINC complex		Nucleus, INM	Altered expression deforms nuclei and changes nuclear stiffness	[43,172,173]
Mammals	Lamin B1/ B2			INM		
Mammals	Sun2	SUN protein	Involved in regulation of nuclear shape, movement and positioning	INM	Overexpression deforms nuclei into a flower-shaped lobular structure	[164]
Mammals	Nesprin1	KASH protein		ONM	Misshaped nuclei, low circularity, changes in lamina structure	[89,174]
Mammals	Nup53, Nup93	Nucleoporins		NE, NPC	Abnormally shaped nuclei	[111]

## 4. Nuclear shape alterations

### Altered nuclear shape during physiological processes

Though a nucleus typically holds a firm shape, the shape can change in response to internal and external stimuli. Nuclear shape normally changes during periods of cell division, migration, development and apoptosis (Figure 1). In cells undergoing open mitosis, the NE disassembles into vesicles and chromosomes are released into the cytoplasm for the duration of M-phase. In cells undergoing closed mitosis, the generally spherical/oval nucleus is stretched into various shapes until it finally splits into two spherical/oval nuclei [22]. Shifting budding yeast cells to a less preferred carbon source also results in reduced circularity [22]. Migration of mammalian cells through confined spaces affects the geometry of the entire cell and its organelles, including the nucleus [144–146] (Figure 1(c)).

Nuclear movement is integral for progress of a cell through development/differentiation, fertilization, and response to environmental cues such as light in plants. During these phases, the nucleus may undergo reorganization of its internal compartments. Blocking movement or shape changes may arrest the proper course of events in the cell. For example, a deficiency of WPP domain interacting tail-anchored proteins, WIT1 and WIT2 in *A. thaliana* results in nuclei with impaired movement as well as an abnormally round shape [147,148]. In *Drosophila*, failure to adopt ellipsoidal nuclear morphology during cellularization results in significantly altered gene expression [24]. These observations support the idea that alterations in nuclear shape are correlated with progression of essential processes like DNA repair, signaling, cell health and homeostasis; however, it is not clear whether distortion of nuclear shape is a cause or consequence of a change in the physiological status of a cell.

### Lamin-dependent modulation of nuclear shape

The expression of lamins is tightly regulated in a cell. The relative amounts of lamin A/C and lamin B impact nuclear flexibility and genomic plasticity. The levels of lamins in a cell are modulated during development and differentiation. For example, mesenchymal stem cells alter the lamin composition and nuclear shape to regulate bone tissue homeostasis. Lamin A levels remain low during early development but increase during osteogenic differentiation [149].

In many instances, lobed nuclei are found in migratory cells [150]. The lower levels of lamin A/C in neutrophils have been linked to their more pliable and fragile nuclei and their shorter life span [11,151]. Reduction in levels of Lamin B receptors in neutrophils results in cells with lower migration rate and rounded nuclei [152]. During cardiac development, nuclear shape and movement of epicardial cells is dependent on lamin B1 [153]. Lb-1 null mouse embryos have a defective myocardial development due to delay in changes in nuclear morphology that result in untimely gene expression and migration behavior. Lamin B1 is also important for supporting nuclear shape in neurons during neuronal migration and its reduced expression impairs brain development which involves neuronal migration [154,155]. These examples illustrate that lamins affect nuclear migration by modulating the elasticity of nucleus [156]. However, altering the lamin profile also alters genome organization, which, in turn, changes expression pattern of genes involved in the various physiological processes and nucleo-cytoskeletal interactions [157,158].

### Pathological consequences of altered nuclear shape

**Figure 3.**
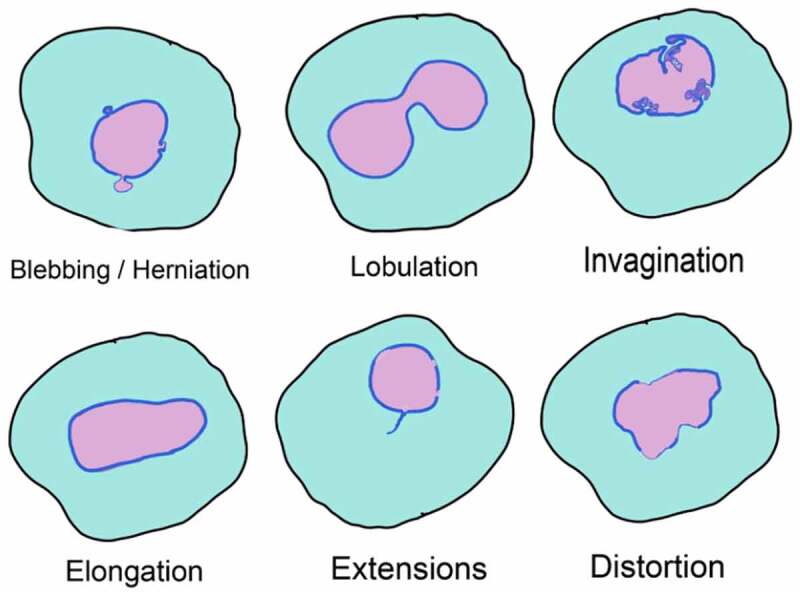
Nuclear shape abnormalities

There is growing evidence of association between nuclear organization defects and several human diseases. These defects are distinct from the morphological alterations of nucleus seen during organismal aging in humans, *Drosophila* and *C. elegans* (Figure 3). This association is clearly established in genetic and metabolic disorders associated with mutations in lamin genes. For example, patients of Hutchinson-Gilford Progeria Syndrome and mandibuloacral dysplasia show cells with aberrant nuclear morphology [7,8]. Nuclear dysmorphia and lobulation is also seen in various cancers such as lung and adenocarcinomas [9, reviewed in 159]. In addition to defects in lamins, loss-of-function mutations in other nuclear proteins such as MAN1, Nesprin-1, Emerin and Torsin1 also result in diseases characterized by abnormal nuclear morphology. These diseases, broadly referred as nuclear envelopathies, include osteopoikilosis, sclerosing bone dysplasias, cerebellar ataxia, X-linked Emery-Dreifuss muscular dystrophy, and DYT1 dystonia [160,161]. Aberrant distribution of NPCs as a result of shape abnormalities has also been implicated in several human pathologies, including autoimmune diseases, viral infections, cardiomyopathies and various cancers .

A graphical representation of various kinds of nuclear shape (magenta] abnormalities. The nuclear contour (blue) differs with the kind of morphological alteration. INM and ONM protein distribution can vary greatly between these defects.

Evagination or excessive outward growth of either INM or ONM or both can result in a bleb like nuclear growth. The size and origin of such NE out growth or herniation can depend on various factors. Some of these mechanisms are described in Thaller and Lusk, 2018. Blebs are also often associated with regions of NE deficient in lamins and therefore prone to rupture.

NE can show invaginations, INM/ONM projecting inwards. Such structures are also termed ‘nucleoplasmic reticulum’ and can originate either at the INM or cytosol. In plants, placental cells in Lilium ovaries have infoldings of INM continuous with the ER lumen, while onion epidermis and tobacco invaginations originate in the cytoplasm [21].

NE protrusions that result in compartmentalization of the nucleus can give a lobular appearance to the nucleus. Lobed nuclei are generally fragile and aid in mobility. Lobed nuclei are often characterized by low lamin A/C content.

An imbalance of forces along the nuclear axes can cause elongation of a quasi-round nuclei. Similarly, lack of factors that maintain a normally elongated nuclei can result in rounding of the nucleus. Sometimes, nucleus can get abnormally shaped without falling into these defined deformities and form ‘tail-like’ membrane extensions or distorted NE.

**Figure 4.**
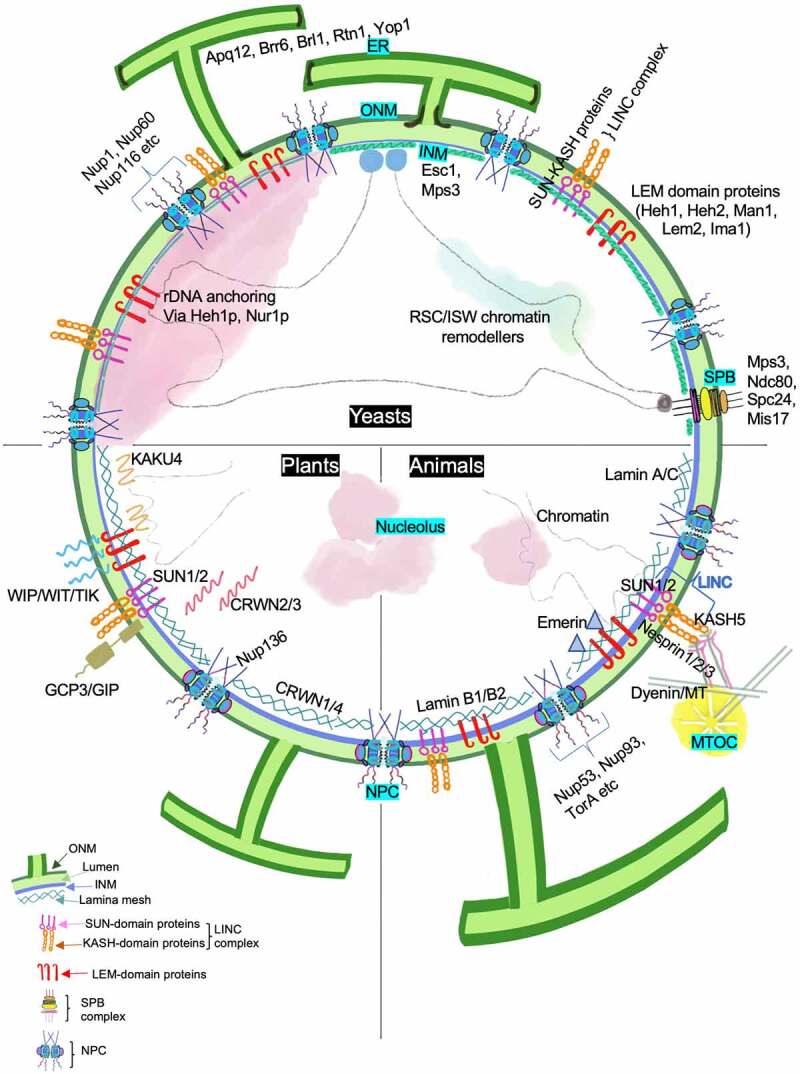
Factors contributing to the maintenance of nuclear shape. The figure summarizes various components that contribute toward regulation of nuclear shape in yeast, plants and animals. Proteins that play a critical role are marked along the associated nuclear sub-compartment and associated organelle. The NE is a double membrane lipid bilayer. The INM has a protein composition that differs from the ONM, with the latter being continuous with ER. Proteins at the INM associate with chromatin and this DNA-protein interaction contributes to overall nuclear stability. Other conserved nuclear compartments are chromosomal territories, nucleolus, telomeric foci and NPCs. In yeast, the SPB complex is INM associated and diametrically opposite to the nucleolus unlike the cytosolic MTOC in animals and plants. Nucleolus is also found at the nuclear periphery in yeast where INM proteins Heh1 and Nur1 anchor the rDNA. On the other hand, the nucleolus in plants and animal cells is away from nuclear periphery, in one or multiple spots

Nuclear blebbing is one of the most common and prominent nuclear deformities observed in various deletion mutants or pathological conditions. Altered nuclear morphology associated with viral infection has been used as a diagnostic measure. For example, flower-like lobulated nuclei are used to diagnose adult T-cell leukemia caused by human T-cell leukemia virus type 1 [162]. HIV infection of terminally differentiated macrophages produces nuclear herniations that are induced by the HIV-1 Vpr protein [163]. The overexpression SUN2 induces lobulation of nuclei, which blocks infection by HIV by affecting early events between reverse transcription and viral entry into nucleus [164]. Nuclear blebs have also been reported in macrophages infected with *Mycobacterium tuberculosis* [165].

These observations reflect the potential of nuclear morphology to affect multiple processes in the cell and reflect its disease status in some cases. Understanding signals which regulate the nuclear shape under normal conditions will provide clues to decipher mechanisms that deform and reshape the nucleus under physiological and pathological conditions.

## 5. Outlook

We have reviewed the diversity in nuclear shape under both physiological and pathological states. Some broad conclusions can be drawn from the research done so far. Firstly, chromatin packaging constraints determine nuclear shape, especially in cells like sperms. Secondly, change in nuclear shape is important for chromosome segregation and nuclear division in nuclei undergoing closed mitosis. Thirdly, it appears that in some cell types the mechanical flexibility provided by a softer, less spherical nucleus is important in development and movement through narrow spaces. Finally, study of changes in nuclear morphology holds potential in the diagnosis and perhaps the treatment of pathologies associated with factors affecting nuclear shape.

How does nuclear shape affect cellular function and, conversely, how do cellular processes affect nuclear morphology? Does a change in nuclear shape alter genome function or does altered genome function lead to shape changes? This question may be answered by developing additional model systems, especially simple single-celled organisms, which are firstly, not constrained by the complexity associated with requirements for multicellular development, and secondly, presumably free of redundancies built into genomes of higher organisms. For instance, the lower eukaryote *C. elegans* has a single lamin gene that is essential for embryonic viability [49], whereas no lamin is individually essential for viability in mammals as they have multiple lamin encoding genes. Simple unicellular model systems may be more amenable to approaches where one could perturb NE shape, chromatin localization, organization of internal components; separately and individually. These models will have to be examined to identify and study conserved players known to be active in determining nuclear shape and nuclear organization. One would require extensive use of bioinformatic tools and development of microscopy-based approaches for these systems.

It is clear that 3D organization of chromatin is important to bring regulatory elements together and loss of this organization does have consequences for gene expression. However, the role of several non-membrane-bound compartments in the nucleus like Cajal bodies that function in the biogenesis of small RNA, or nuclear speckles that store and recycle splicing factors, needs further investigation. These bodies are thought to self-assemble in a concentration-dependent manner and could be phase-separated as well [166]. Changing nuclear shapes could affect the local concentration of the factors that contribute to these bodies and hence may change the number and /or the composition of the nuclear body, thereby affecting its function. It would be interesting to investigate the structure and function of nuclear sub-compartments in nuclear shape mutants.

Lastly, understanding the interplay between the various regulators of nuclear shape would be critical to differentiate between direct consequences versus indirect consequences of nuclear shape alterations. Lipid composition, nuclear transport and NE components would be the mediators of nuclear shape and nuclear organization, but the regulators could be dynamic. Nutritional status could change lipid availability that can have consequences on NE composition that affects the INM protein targeting [126,167]. Future studies should address all these aspects and provide insights that will allow us to develop a holistic understanding of whether nuclear form affects function and if yes, how.

However, while various factors – such as components of the NE, elements inside the nucleus, lipid metabolism and nuclear transport – affecting nuclear shape have been identified in several eukaryotes, the details of how these pathways operate singly and in combination are not known. A tremendous amount of work needs to be done to answer key questions such as why is it important for a cell to maintain or change the shape of its nucleus, and how these changes are resisted or initiated.

## Disclosure of Potential Conflicts of Interest

No potential conflict of interest was reported by the author(s).
